# Parental Digital Tracking of Adolescents' School Performance, Location, and Finances: Widespread, Often Accepted, and Linked to Overprotection

**DOI:** 10.1002/jad.70229

**Published:** 2026-07-20

**Authors:** Savannah Boele, Anne Bülow, Loes Keijsers

**Affiliations:** ^1^ Erasmus School of Social and Behavioral Sciences Erasmus University Rotterdam Rotterdam the Netherlands

**Keywords:** adolescence, digital tracking, monitoring, parental knowledge, parenting, surveillance

## Abstract

**Introduction:**

Digital tracking tools are now at parents' fingertips, raising concerns about infringement on adolescents' autonomy, privacy, and well‐being. Despite growing concerns about parental digital surveillance, empirical research beyond location tracking remains limited. This study examined parental digital tracking across three key domains: school performance, location, and finances.

**Methods:**

This is a multi‐informant, cross‐sectional survey study (2024) including 525 Dutch parent–adolescent dyads (adolescents: *M*_age_ = 14.1, range = 11–18; 53% female; parents: *M*_age_ = 46.7, 81% mothers). A second sample (*N* = 175 dyads; 2019) tested replication and changes over time.

**Results:**

In 2024, nearly all parents (99.6%) tracked at least one domain. Most adolescents accepted tracking, though location tracking was accepted less. Younger, less educated parents and mothers were tracked more frequently, particularly in school performance. Adolescents following a lower secondary educational track were tracked more frequently. Younger adolescents and boys experienced more frequent school tracking, whereas older adolescents were tracked more frequently in location. Digital tracking was unrelated to adolescent well‐being (e.g., anxiety symptoms) but positively associated with parental control‐ and fear‐related factors (e.g., overprotection, dangerous worldviews). School and location tracking increased over the past 5 years, as did adolescents' acceptance of tracking.

**Conclusions:**

Parental digital tracking appears a common tool for many parents today, and its frequent use may reflect parental insecurities, with no clear link to adolescent maladjustment. Longitudinal research is needed to examine whether digital tracking reassures parents or reinforces fears, which might undermine parents' ‐ and potentially also adolescents' ‐ well‐being.

## 1 Introduction

Digital tracking tools allow parents to passively monitor nearly every aspect of their child's life, from physical location and school performance to financial expenses (Davis et al. 2024; Gabriels 2016). Such tools may be appealing because they offer effortless, continuous, and accurate access to real‐time and historical information, surpassing information resulting from conversation‐based monitoring (Pelham et al. 2024; Stattin and Kerr 2000). However, the ability to digitally surveil adolescents – potentially without their consent or awareness ‐ raises concerns about overprotection, mistrust, privacy violations, and constraints on autonomy (Davis et al. 2024; Gabriels 2016; Gelman et al. 2021). Despite these concerns, empirical evidence remains limited and largely focused on location tracking (see Davis et al. 2024 for review). Prior research reveals that roughly half of U.S. parents report tracking their adolescents' location (Burnell et al. 2023), a practice that has been associated with overprotective parenting (Chase et al. 2024). However, much less is known about digital tracking across other domains. This preregistered, multi‐informant study (*N* = 525; 2024) investigated parental digital tracking across three domains ‐ location, school performance, and finances ‐ and compared findings with a 2019 sample (*N* = 175).

### 1.1 From Dialogue to Data: Conversation‐Based Monitoring Versus Passive Digital Tracking

As adolescents seek greater independence with age (Branje 2018), they strategically manage information and gradually disclose less about their whereabouts and functioning to their parents (Darling et al. 2007; Lionetti et al. 2018; Smetana 2024). In response, parents seek information (Stattin and Kerr 2000). Though the ‘traditional’ monitoring literature (see Table 1) has acknowledged passive strategies, such as being informed by other parents (Waizenhofer et al. 2004), more attention has been paid to active strategies, such as parental solicitation and control, and adolescent information management strategies, such as adolescent disclosure and secrecy (Pelham et al. 2024; Smetana et al. 2024). A key finding has been that adolescents' disclosure is an important source of parental knowledge and predicts better adolescent adaptation — and vice versa, as better‐adjusted adolescents also tend to disclose more to their parents (Kapetanovic et al. 2019; Stattin and Kerr 2000).

**Table 1:** The characteristics of traditional monitoring versus digital surveillance.

	Traditional monitoring (conversation‐based)	Digital Tracking (data‐driven)
Source of information	Parent‐adolescent communication (e.g., solicitation, disclosure)	Digital tools (e.g., GPS trackers, online school portal, banking apps)
Accuracy	Subjective and may be incomplete or inaccurate.	Objective and precise.
Context	Rich in emotional and motivational cues.	Lacks emotional or motivational cues.
Timeliness	Pre‐ or post‐activity: Situational or delayed^a^ (typically one activity)	Instant access to real‐time and/or (aggregated) historical data.
Parental effort	Active: asking questions, demanding information, inquiring.	Less active: watching, surveillance. Minimal effort and independent of the relationship.
Adolescent Agency	Active (e.g., disclosing, lying, or withholding information).	Passive or none (depending on app/portal settings).
Adolescent Consent	Implicit: actively managing information.	Not always required (depending on app/portal settings).
Adolescents' Awareness	Usually aware (except: indirect solicitation; snooping).	Often unaware of when or how tracked.

^a^ Information could be more delayed when obtained through online communication.

The rise of technology has provided parents with new possibilities to passively obtain information. Digital tools like GPS trackers, online school portals, and banking apps allow parents to passively monitor their adolescents' location, school performance, and financial activities. Compared with conversation‐based monitoring, digital tracking provides effortless, accurate, and both historical information (e.g., locations in the past hour, overview of school grades, spending overviews) and real‐time insights (e.g., current location, newest school grade, current bank account balance) but lacks emotional and contextual cues (e.g., emotions or reasons behind a poor grade) and may also operate without adolescents' active participation or consent. Overall, digital tracking seems a highly one‐sided dynamic, where information flows effortlessly to parents, often without adolescent input or consent. Given the developmental importance of adolescent agency and autonomy, both of which may be compromised in digital tracking, it is essential to investigate how parenting becomes datafied.

### 1.2 Which Domains Are Being Tracked?

Parental (digital) monitoring is inherently domain‐specific (Pelham et al. 2024). Yet, little is known about how tracking extends beyond location to other domains. Studies tentatively suggest that the use of GPS tracking among parents has increased over the past decade (Davis et al. 2024). In a recent U.S. study, 51% of 646 parents reported tracking their adolescent's location, ranging from once in a while to always (Burnell et al. 2023). Parents today also have access to online school portals and to banking applications, for example. Parents may have different motivations to track distinct domains. For example, they may track location out of safety concerns, finances to foster responsibility, and school performance to stay informed about academic progress, each potentially carrying distinct implications for adolescent development. To better understand the scope of contemporary parental digital surveillance, we examined how often parents digitally track in three domains: location, school, and finances (RQ1), and whether some parents digitally track broadly across domains while others track selectively or not at all (RQ2).

### 1.3 In Which Families Does Parental Digital Tracking Occur More Frequently?

Previous research has extensively shown that demographic characteristics are related to traditional “offline” parental monitoring (Pelham et al. 2024; Racz and McMahon 2011). Adolescent girls are more likely to be monitored than adolescent boys, and mothers tend to be more knowledgeable about their child's whereabouts than fathers. Moreover, parental monitoring efforts tend to decline during adolescence (Lionetti et al. 2018), and higher levels of knowledge and monitoring are observed in families with lower socioeconomic status (Racz and McMahon 2011). Whether such patterns translate to digital tracking remains unclear (Davis et al. 2024): higher parental educational attainment has been associated with the use of location tracking (Chase et al. 2024), whereas no significant link has been found between SES and tracking frequency (Burnell et al. 2023). We examined whether such between‐family differences translate to digital tracking and explored how parents' (RQ3) and adolescents' (RQ4) demographic characteristics (i.e., sex, age, and education) relate to the frequency of parental digital tracking across the domains.

### 1.4 Do More Anxious and Neurotic Parents Digitally Track More Frequently?

Parental anxiety about their child's safety and functioning may be a key driver of digital tracking (Davis et al. 2024). Such parental anxiety may have different determinants. Parents with higher levels of *general anxiety*, characterized by persistent, excessive worry across various domains, may be more prone to worry about their children (Segrin et al. 2013), potentially relating to more frequent digital tracking (across domains) (H1). Additionally, the trait *neuroticism* (i.e., the tendency to experience and the inability to cope with negative emotions and situations; Caspi et al. 2005) has been related to more overprotective and less autonomy‐supportive parenting (Prinzie et al. 2009), and might therefore also relate to more frequent digital tracking (across domains) (H2).

### 1.5 Are Parents Who Frequently Use Digital Tracking More Supportive or Overprotective?

As digital tools offer parents continuous access to their children's lives, questions should be raised about whether this access fosters supportive involvement or tips over into overprotection. Prior research suggests that parents who are more knowledgeable about their child's whereabouts and actively seek information tend to be more involved and more autonomy‐supportive (Baudat et al. 2020; Pelham et al. 2024). However, monitoring may also tip over into violations of privacy (Hawk et al. 2008), and location tracking use has been associated with overprotective parenting (Chase et al. 2024). To clarify whether frequent tracking reflects involvement or overprotection, we examine its association with emotional support (RQ5), autonomy support (RQ6), and overprotection (RQ7).

### 1.6 Is Digital Tracking Accepted by Adolescents?

Adolescents may view digital tracking as invasive and autonomy‐threatening, especially when it occurs without their consent or awareness. However, adolescents may see it as a normal and acceptable aspect of contemporary parenting, as they are being raised in a ‘data‐driven algorithmic culture’, which embraces data‐driven information to understand ourselves and others (Gabriels 2016). Perceived acceptance may also depend on the domain (Darling et al. 2008; Van Petegem et al. 2017). Tracking that concerns prudential (e.g., safety and health) or conventional (e.g., social norms) domains may be seen as more acceptable than tracking in personal domains, which adolescents typically consider private and outside parental authority (Darling et al. 2007; Smetana 2024; Smetana et al. 2005). School performance is generally experienced as a less personal matter than finances or whereabouts (Darling et al. 2007). To shed light on how adolescents feel about being tracked by parents, we described adolescents' perceptions of their acceptance of digital tracking (RQ8).

### 1.7 How Is Parental Digital Tracking Relating to Adolescents' Well‐Being?

The shift from interaction‐based parental knowledge to data‐driven methods appears to reduce adolescent agency and may therefore have psychological consequences. Being digitally tracked, especially frequently and possibly without consent, might feel invasive (Davis et al. 2024; Gelman et al. 2021) and undermine adolescents' need for autonomy and competence by signaling distrust or constant evaluation (Wood et al. 2003). As a result, frequent tracking might contribute to lower self‐esteem and poorer mental well‐being in adolescents (Šutić et al. 2025; Zhang and Ji 2024). To explore these risks, we tested the hypotheses that higher tracking frequency relates to more autonomy frustration (H3), depressive (H4a), and anxiety symptoms (H4b), and tracking of school performance to more school pressure (H5).

### 1.8 Current Study

Digital technology has provided parents with new opportunities to passively monitor their adolescents, raising novel fundamental questions about contemporary parenting and adolescent development. To examine this emerging parenting practice of digital surveillance, the current preregistered study (https://osf.io/xvf5h) analyzed cross‐sectional data (from October 2024) from 525 parent–adolescent dyads, addressing exploratory research questions (RQ1–8) and hypotheses (H1–5). A second sample of 175 dyads collected in 2019 was included to test for replication and potential changes over time.

## 2 Materials and Methods

### 2.1 Participants

In total, 548 parent‐adolescent dyads registered in 2024 to participate in the PARADOx‐project (https://osf.io/zfcmd). Dyads were included in the current study if data were available for parental digital tracking, resulting in an analytical sample of 525 dyads. The adolescents had a mean age of 14.1 years (SD = 1.6, range = 11–18), with 52.6% having recorded sex as female at birth, 45.7% as male, and 0.2% as other, and the majority were born in the Netherlands (95.4%). Adolescents' educational tracks ranged from pre‐vocational or vocational training (18.1%) to pre‐university education (43.2%). Parents (*M*_age_ = 46.7 years, *SD*_age_ = 4.7, age range = 31–61) were mostly biological mothers (81%) or fathers (18%) and often cohabited with the other biological parent (80%). Most reported equally sharing caregiving responsibilities (67%). Parents' educational backgrounds ranged from secondary school (3.4%) to university (33.1%). Detailed demographic information is provided in Table A1 of the Supporting Information A.

### 2.2 Procedure

Data of the current study is based on the baseline questionnaire of the PARADOx‐project (more information https://osf.io/zfcmd). Recruitment started in late August 2024. Adolescent‐parent dyads were primarily recruited through Dutch high schools in both rural and urban areas. Participants from previous similar studies were also invited (Boele et al. 2024), and peer‐to‐peer recruitment was implemented. Eligible dyads included adolescents aged 12–17 who saw their parent at least three times per week and spoke Dutch. Interested participants accessed study details, a short explanatory video, and online consent forms. Active informed consent was obtained from both parents and adolescents, and parental consent for adolescents under 16. On October 13, 2024, participants received a personal survey link. Each received €5 upon completion. Procedures were approved by the DPECS Research Ethics Review Committee at Erasmus University Rotterdam (ETH2324‐0156).

### 2.3 Measures

#### 2.3.1 Digital Tracking of School Performance, Location, and Finances

Parents completed three items assessing how often they digitally track their adolescent's school performance, location, and financial activities (see items in Table 2). Each item was rated on a 5‐point scale: 1 (*Never*), 2 (*A few times per year*), 3 (*A few times per month*), 4 (*A few times per week*), 5 (*A few times per day*). For financial tracking, two additional options were available: “I don't know” and “My child does not have their own bank account”, which were handled as missing values.

**Table 2:** Exploratory factor analysis and inter‐item correlations of the digital tracking items.

Item No.	Item	Factor loadings	*h* ^2^	Inter‐item correlations	
				1	2
1.	How often do you digitally monitor school‐related information (e.g., grades, homework, or attendance via Magister)?	0.47	0.22	—	**—**
2.	How often do you digitally track your child's location (e.g., via Find My iPhone or a GPS tracker)?	0.42	0.17	0.20*	—
3.	How often do you check the money in your child's bank account (e.g., via a banking app)?	0.62	0.40	0.29*	0.26*

*Note: h* ^2^ = communalities, representing the proportion of each item's explained variance by the factor.

* *p* < 0.001.

#### 2.3.2 Parental Emotional Support

Parents and adolescents reported on the official short form of the Network of Relationships Inventory ‐ Social Provisions Version (NRI‐SPV; Furman and Buhrmester 1985), assessing seven aspects of support (e.g., affection, enhancement of worth Weiss 1974). The items (e.g., “how much do you/does your parent really care about your child/you?”) were rated on a 5‐point scale from 1 (*little or none*) to 5 (*the most*). Internal consistency was acceptable to excellent (*α* = 0.74 parents; *α* = 0.85 adolescents).

#### 2.3.3 Parental Autonomy Support

Parents and adolescents reported on the Perceptions of Parents Scale (POPS; Grolnick et al. 1991), which assesses the extent to which parents encourage their child to follow their own interests and values (Vansteenkiste et al. 2005). The POPS consists of seven items (e.g., “My parent encourages me to decide things for myself”), with a 5‐point response scale from 1 (*not true at all*) to 5 (*completely true*). Internal consistency was acceptable (*α* = 0.68 parents; *α* = 0.72 adolescents).

#### 2.3.4 Parental Overprotection

Parents and adolescents completed the short version of the Multidimensional Overprotective Parenting Scale (S‐MOPS; Chevrier et al. 2023), a 16‐item measure assessing anxious (10 items; e.g., “My parent treats me like a small child”) and ego‐enhancing overprotection (6 items; e.g., “My parent thinks that everything I do is extraordinary”). Items were rated on a 5‐point scale from 1 (*not at all true*) to 5 (*completely true*). Internal consistency was acceptable (*α* = 0.77 parents; *α* = 0.76 adolescents).

#### 2.3.5 Parental Anxiety Symptoms

Parents completed the Generalized Anxiety Disorder Scale (GAD‐7; Spitzer et al. 2006), a seven‐item measure of generalized anxiety symptoms over the past 2 weeks (e.g., “Feeling nervous, anxious, or on edge”). Items were rated from 0 (*not at all*) to 4 (*nearly every day*). Internal consistency was excellent (*α* = 0.85).

#### 2.3.6 Parental Neuroticism

Parents completed the Neuroticism subscale of the Big Five Inventory‐2‐S version (Denissen et al. 2020). The subscale includes six items (e.g., “I worry a lot”), rated on a 5‐point scale from 1 (*totally disagree*) to 5 (*totally agree*). Internal consistency was good (*α* = 0.78).

#### 2.3.7 Adolescents' Acceptance of Parental Digital Tracking

Adolescents rated how acceptable it was for their parents to digitally monitor or access certain platforms, using items adapted from the Legitimacy Beliefs for Parental Authority Scale (Kuhn and Laird 2011; Smetana and Daddis 2002). The statement was, “I find it okay that my parent(s) digitally monitor or access…,” followed by three items: school, GPS/iPhone tracker, and bank account app, rated from 1 (*Not okay*); 2 (*Okay a little*); 3 (*Totally okay*).

#### 2.3.8 Adolescent Autonomy Frustration

Adolescents completed the Basic Psychological Need Satisfaction and Frustration Scale (Chen et al. 2015). The Autonomy Frustration subscale consists of 8 items (e.g., “Most of the things I do feel like ‘I have to’”). The response scale ranges from 1 (*not at all true*) to 5 (*very true*). The scale shows excellent internal consistency (*α* = 0.82).

#### 2.3.9 Adolescent Depressive Symptoms

Adolescents completed the Reynolds Adolescent Depression Scale – Short Form (RADS‐2‐SF; Milfont et al. 2008; Reynolds 2005), a 10‐item measure of depressive symptoms (e.g., “I felt sad”) experienced over the past 2 weeks. Items were rated from 1 (*almost never*) to 4 (*often*). Internal consistency was excellent (*α* = 0.88).

#### 2.3.10 Adolescent Anxiety Symptoms

Adolescents completed the 9‐item General Anxiety subscale of the Screen for Child Anxiety Related Emotional Disorders (SCARED; Birmaher et al. 1997), rating how often they experienced each symptom (e.g., “I was worried about how well I was doing things”) in the past 3 months (0 = *never* to 2 = *often*). Internal consistency was excellent (*α* = 0.88).

#### 2.3.11 Adolescent School Pressure

Adolescents indicated how much school pressure they experienced in the past week experienced with one item (Boele et al. 2024): “To what extent did you feel pressured by schoolwork?”. The item was rated from 1 (*never*) to 5 (*always*).

#### 2.3.12 Preregistered Statistical Analytical Plan

We preregistered our statistical analysis plan (https://osf.io/xvf5h). Because residuals were not normally distributed (as examined by Q–Q plots), robust regression with M‐estimation was used to reduce the influence of outliers and violations of normality and homoscedasticity (Andersen 2008). Statistical significance was set at *α* = 0.05. All analyses were conducted in R (version 4.4.0). Data and R code can be viewed online: https://osf.io/znu9w/.

## 3 Results

The descriptive statistics of all study variables and the bivariate between‐person correlations are reported in the Supporting Information A (Tables A2, A3).

### 3.1 Exploratory Factor Analysis of Parental Digital Tracking

To examine whether parents track across domains similarly or selectively (RQ2), we conducted an EFA. The EFA with the three digital tracking items showed factor loadings ranging from 0.42 to 0.62 (Table 2). Together, the items explained 26% of the variance in the factor. The eigenvalue is 0.79, and the Cronbach's alpha is 0.48, indicating low internal consistency among the items. These findings provide little support for a single underlying construct, suggesting that these tracking behaviors are domain‐specific. Subsequent analyses were therefore conducted separately for each domain.

### 3.2 Sample Frequencies of Parental Digital Tracking

To address how often parents digitally track across domains (RQ1), we examined sample frequencies. Almost all 525 parents (99.6%) reported tracking their adolescent in at least one domain (Table 3). Tracking school performance was most common (96.4%), followed by financial activities (90.1%) and location (65.7%). Tracking frequency varied widely across parents, from *several times a year* to *several times a day*. However, only 7.8% tracked all domains equally frequently, indicating variation across domains within the same parents. Figure B1 of Supporting Information B shows descriptive clusters.

**Table 3:** Sample frequencies of self‐reported parental digital tracking behaviors (*N* = 525).

	Domain of digital tracking			
Response	School	Location	Finances	Similar frequency across domains
Never tracked	19 (3.6%)	180 (34.3%)	52 (9.9%)	2 (0.4%)
Engages in tracking	506 (96.4%)	346 (65.7%)	473 (90.1%)	—
*Self‐reported frequency*				
1. Several times a year	82 (15.6%)	109 (20.8%)	176 (33.5%)	9 (1.7%)
2. Several times a month	172 (32.8%)	83 (15.8%)	**228 (43.4%)**	9 (1.7%)
3. Several times a week	**219 (41.7%)**	**135 (25.7%)**	63 (12.0%)	**21 (4.0%)**
4. Several times a day	33 (6.3%)	18 (3.4%)	6 (1.1%)	0 (0.0%)

*Note:* Bold values indicate modus (i.e., most chosen answer).

### 3.3 Robust Regression Models Predicting Tracking Frequency

To understand which parents engage more frequently in digital tracking (RQ3), we first explored parent demographic characteristics (Model 1; see Table 4). Digital tracking frequency varied by parent demographics: younger parents engaged more frequently in digitally tracking school performance and financial activities (βs = −0.14 to −0.15, *p*s ≤ 0.004). School performance was more frequently tracked by mothers compared to fathers (*β* = −0.31, *p* = 0.008) and by parents with lower or middle levels of education compared to highly educated parents (*β* = −0.22, *p* = 0.038).

**Table 4:** Results of robust regression models predicting frequency of digital tracking.

	Digital tracking behaviors		
	School	Location	Finances
	β	β	β
Model 1: Parent characteristics			
Age	−0.15**	−0.02	−0.14**
Sex (0 = female (81.9%), 1 = male (18.1%))	−0.31**	−0.23	−0.16
Educational track (0 = low and middle (22.0%), 1 = high (78.0%))	−0.22*	−0.13	−0.16
Model 2: Adolescent characteristics			
Age	−0.20***	0.10*	−0.08
Sex (0 = female (53.4%), 1 = male (46.4%))	0.29**	−0.06	0.17
Educational level (0 = low and middle (53.5%), 1 = high (46.5%))	−0.24***	−0.19*	−0.48***
Model 3: Parent well‐being and personality			
Parental anxiety	−0.04	0.03	−0.01
Parental neuroticism	0.06	0.05	0.02
Model 4: Parent‐reported parenting			
Emotional support	0.18***	0.08	0.10*,^a^
Autonomy support	−0.03	0.03	−0.05
Overprotection	0.14**	0.18***	0.13*
Model 5: Adolescent‐reported parenting			
Emotional support	0.09	0.05	0.07
Autonomy support	−0.09	−0.01	−0.11*,^b^
Overprotection	0.19***	0.06	0.16***
Model 6: Adolescent well‐being			
Anxiety symptoms	−0.05	−0.00	−0.17*,^b^
Depressive symptoms	−0.08	0.07	0.13
Autonomy frustration	0.06	−0.05	−0.05
Model 7: Adolescent school pressure			
Adolescent school pressure^1^	−0.01	—	—

*Note*. Educational level parents: low = secondary school; middle = vocational training; high = (applied) university). Educational tracks adolescents: low = pre‐vocational training; middle = higher general secondary education or vocational training; high = pre‐university or (applied) university.

* *p* < 0.05

** *p* < 0.01

*** *p* < 0.001.

^a^ Effect does not remain significant after controlling for parental age, sex, and education.

^b^ Effect does not remain significant after controlling for adolescent age, sex, and education.

To understand which adolescents are tracked more frequently (RQ4), we subsequently explored adolescent demographic characteristics (Model 2). Younger adolescents were tracked more frequently in the school domain (*β* = −0.20, *p* < 0.001), whereas older adolescents had their location tracked more frequently (*β* = 0.10, *p* = 0.031). Moreover, boys' school performance was tracked more often than girls (*β* = 0.29, *p* = 0.001). Adolescents in higher educational tracks experienced less frequent digital tracking across all three domains (βs = −0.19 to −0.48, *p*s ≤ 0.040).

To test whether more anxious (H1) or neurotic (H2) parents track more, we examined parental personality (Model 3). Contrary to H1 and H2, parental general anxiety and neuroticism were not significantly related to tracking frequency.

To examine whether tracking frequency reflects supportive involvement or overprotection (RQ5–7), parenting dimensions were examined (Models 4 and 5). Overprotective parenting was most consistently associated with tracking frequency, such that more overprotective parents tracked more frequently across domains (βs = 0.13 to 0.19, *p*s ≤ 0.011).

Finally, we tested whether higher tracking frequency is associated with more autonomy frustration (H3), depressive (H4a), and anxiety symptoms (H4b), and school pressure among adolescents (H5; Models 6 and 7). However, no support was found for these hypotheses.

### 3.4 Adolescents' Acceptance of Parental Digital Tracking

To understand how adolescents perceive being tracked by their parents (RQ8), we examined adolescents' acceptance of parental digital tracking per domain. Most adolescents considered tracking of school performance and financial activities an acceptable practice (see Figure 1). In contrast, location tracking was viewed as “not okay” by approximately one‐quarter. Notably, perceptions of acceptance varied across domains within adolescents: only 0.8% rejected tracking in all domains, 3.1% found tracking all domains somewhat acceptable, while 28.2% considered it entirely acceptable to track all domains.

**Figure 1:**
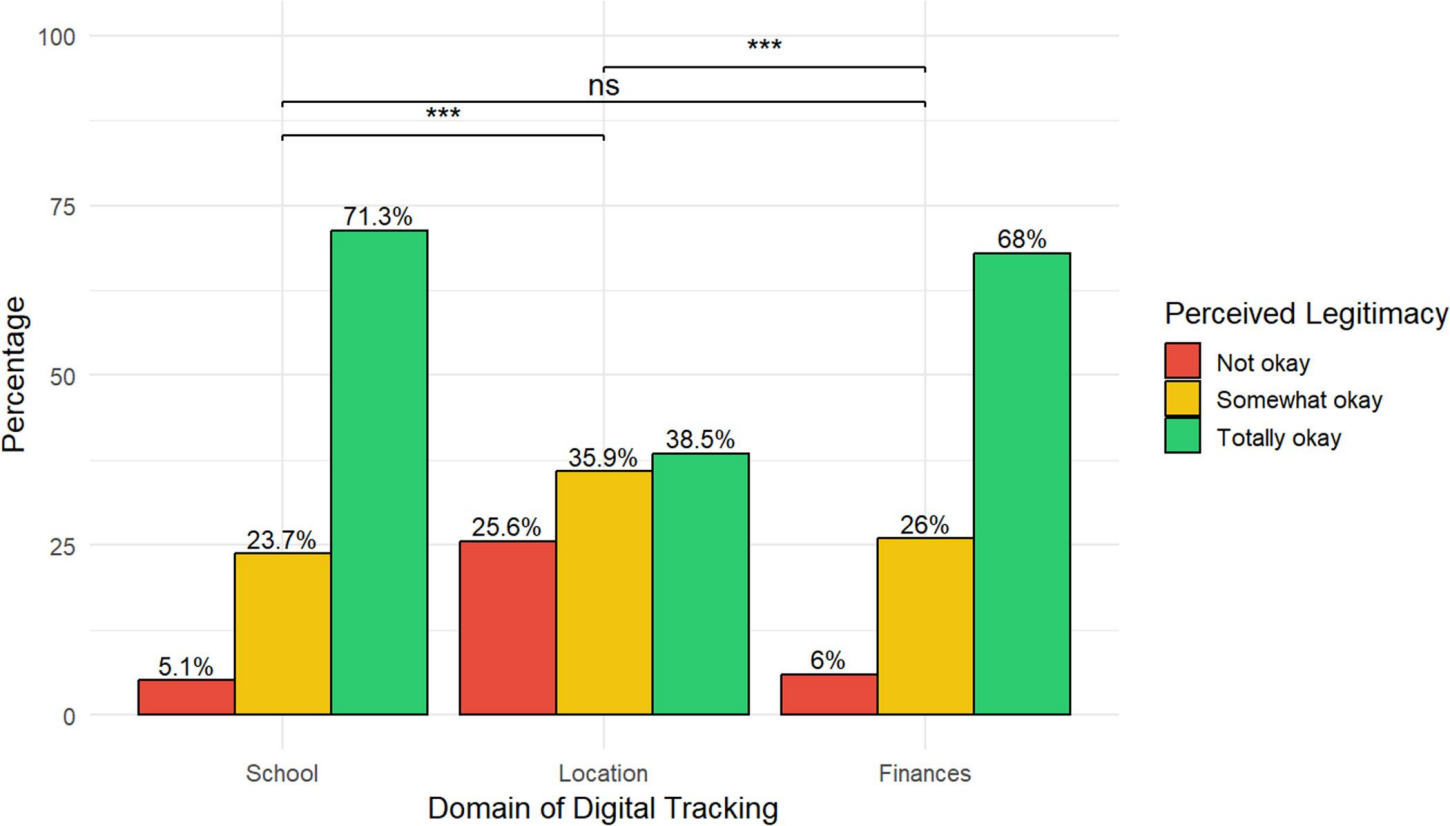
Adolescents' acceptance of parental digital tracking by domain (*n* = 515). *Note:* Friedman's test indicated significant differences in acceptance across domains (χ^2^(2) = 220.8, *p* < 0.001). Location tracking was perceived as significantly less accepted than school tracking and financial tracking (*p*s < 0.001, *r*s = 0.52–0.54); school and financial tracking did not differ significantly (*p* = 0.45). ***p < 0.001, ns = not significant.

### 3.5 Preregistered Replication Study 2 (*N* = 175; 2019–2020)

Sample and results of Study 2 are described in the Supporting Information C and Tables C1–C6. Similar measures were used as in Study 1 (see https://osf.io/xvf5h). In both samples, overprotective parenting was positively related to digital tracking across domains, younger adolescents were more frequently tracked in the school domain, and adolescents following lower educational tracks were more frequently tracked financially (Supporting Information: Table C5). In contrast to Study 1, parent demographic factors were not associated with tracking frequency in Study 2, and parental anxiety symptoms were positively associated with school tracking in Study 2 (but not in Study 1).

### 3.6 Exploratory Non‐Preregistered Analyses With Study 1

#### 3.6.1 Understanding Parents' Motivations: Characteristics Related to Anxiety and Pressure

Given the link between digital tracking and overprotection, we explored whether established predictors of overprotection were also associated with tracking frequency (see Supporting Information D). Parents' intolerance of uncertainty, separation anxiety, and a dangerous worldview consistently emerged as correlates of digital tracking (Table 5). School tracking was uniquely correlated to higher levels of parenting perfectionism. Similar traits in adolescents were unrelated to their parents' digital tracking behaviors, however.

**Table 5:** Exploratory bivariate spearman correlations related to anxiety and pressure.

	Digital Tracking Behaviors		
	School	Location	Finances
	*r*	*r*	*r*
Parent characteristics (*n* = 518 to 521)			
Parenting perfectionism (*α* = 0.81)	0.18***	0.03	0.02
Separation anxiety (*α* = 0.80)	0.10*	0.13**	0.08
Intolerance for uncertainty (α = 0.80)	0.13**	0.10*	0.09*
Threat perception (*α* = 0.80)	0.04	0.13**	0.07
Dangerous worldview (*α* = 0.81)	0.17***	0.17***	0.14**
Optimism (*α* = 0.76)	−0.06	−0.08	−0.08
Adolescent characteristics (*n* = 514)			
Perfectionism (*α* = 0.87)	−0.02	0.03	−0.08
Separation anxiety (*α* = 0.78)	−0.01	−0.02	−0.04
Intolerance for uncertainty (*α* = 0.81)	−0.07	0.08	−0.01

*Note:* Bivariate correlations were calculated to avoid multicollinearity due to the moderate to large correlations among predictors (see Tables D1 and D2 in Supporting Information D).

* *p* < 0.05

** *p* < 0.01

*** *p* < 0.001.

### 3.7 Parent‐Reported Versus Adolescent‐Reported Tracking Frequency

In the follow‐up survey (*n* = 271; April 2025), adolescents reported how often they *believed* their parents digitally tracked them. About half of the dyads agreed on tracking frequency (Figure 2). When discrepancies occurred, adolescents often reported a higher tracking frequency. In 4%–7% of dyads, adolescents believed they were not tracked despite parental reports, and in 3%–12%, the reverse pattern emerged.

**Figure 2:**
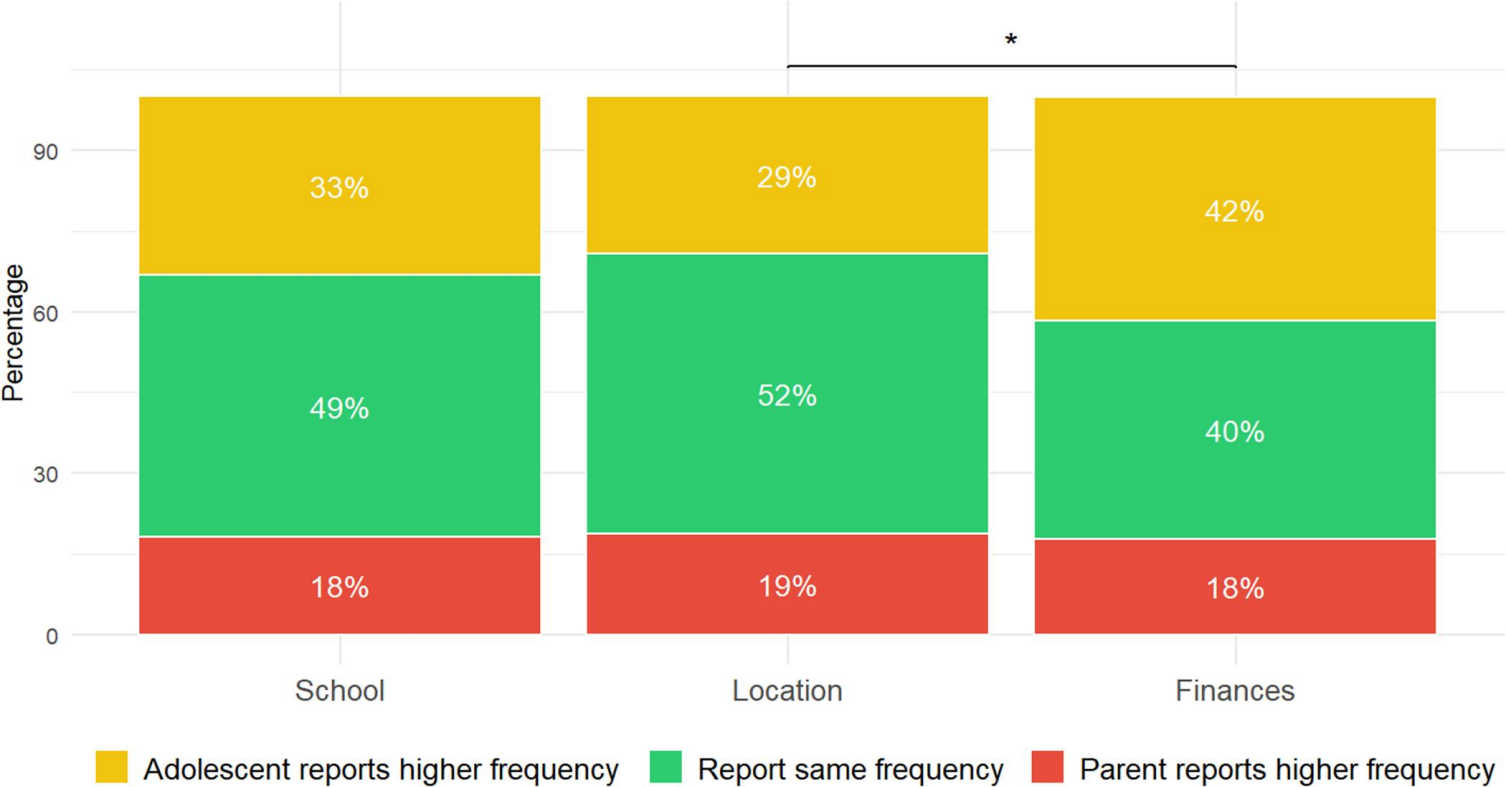
*D*yadic perception of parental tracking frequency (*n *= 271). *Note*: Cochran's *Q* test indicated significant differences in dyadic agreement across domains (Q(2) = 8.01, *p* = 0.018). Agreement was significantly lower for financial tracking than for location tracking (*p* = 0.026); remaining comparisons were non‐significant. *p < 0.05.

### 3.8 Digital Tracking: 2019 Versus 2024

When controlling for sample differences in parent and adolescent age, sex, and education, the ordinal regression analyses (Figure 3) revealed that parents in 2024 reported a higher frequency of tracking school performance (*β* = 0.62, *p* = 0.015) and location compared to parents in 2019 (*β* = 1.47, *p* < 0.001). Adolescents in 2024 viewed tracking of location (*β* = 2.61, *p* < 0.001) and finances (*β* = 0.73, *p* = 0.010) as more acceptable than adolescents in 2019 (Figure 3).

**Figure 3:**
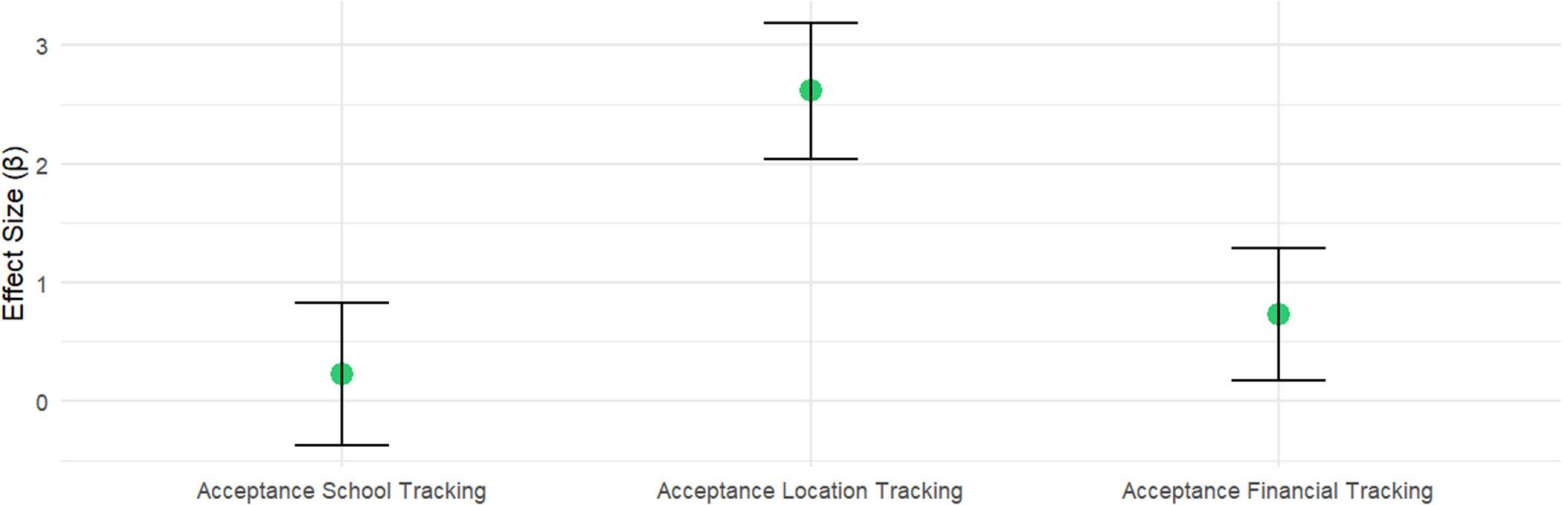
Changes in tracking and acceptance: comparison between 2019 (*N* = 175) and 2024 (*N* = 525) independent samples.

## 4 Discussion

Parents today have a wide range of strategies to stay informed about their adolescents' lives, from physical conversations to online communication and passive digital tracking (Beyens et al. 2024; Pelham et al. 2024; Smetana et al. 2024). This use of digital tracking has sparked concerns of undermining the adolescents' autonomy, privacy, and well‐being (Davis et al. 2024; Gabriels 2016), echoing broader debates on the potential rise and harmful consequences of fear‐driven overprotective (Haidt 2024; Malone 2007) and “paranoid parenting” (Furedi 2001). Despite these concerns, empirical research on parental digital tracking (also called digital surveillance) of “offline” activities remains scarce and has largely focused on location tracking (Davis et al. 2024). To begin addressing this form of digital parenting (Modecki et al. 2022), this cross‐sectional, multi‐informant study among 525 parent–adolescent dyads examined the prevalence and predictors of parental digital tracking across three key domains: school performance, location, and financial activities.

Findings show that, in 2024, nearly all parents (99.6%) engaged in digital tracking, though frequency varied across parents and domains. Most adolescents accepted digital tracking, particularly school performance and financial activities, while location tracking was viewed more critically. Parental (not adolescent) characteristics were associated with tracking frequency, including overprotection, intolerance of uncertainty, and perceiving the world as dangerous. These findings suggest that frequent digital tracking may reflect parents' own anxiety and concerns, rather than issues in adolescent maladjustment.

### 4.1 Digital Tracking Is Domain Specific, Prevalent, and Often Accepted by Adolescents

Digital tracking has become a common tool for many parents today. However, digital tracking appears domain‐specific; parents vary in how frequently they track the different aspects of their adolescents' lives, perhaps depending on the perceived needs or concerns of the parent. School performance was tracked most frequently: nearly half of the parents checked the online school portal several times a week. Location tracking showed the greatest variability: about one‐third of parents did not use it, while others ranged from occasional to weekly use. Our prevalence rate for location tracking of 65.7% falls at the upper end of the 33%–69% range reported across prior studies, most of which were conducted in the U.S. (Davis et al. 2024), which may reflect cultural differences, increases in use over time, or both.

Although location was the least tracked overall, comparisons between independent data from Dutch families collected in 2019 (*N* = 175) and 2024 (*N* = 525) tentatively suggest that it has increased in the Netherlands. This trend aligns with prior studies, collectively suggesting a growing use of GPS‐based tools among parents (for review see Davis et al. 2024). The COVID‐19 pandemic may have contributed to this trend. Dutch parents spent more time with their adolescents during the pandemic, and highly controlling parents became more controlling (Bülow et al. 2021). As adolescents regained independence post‐pandemic, parents may have turned to digital tracking to compensate for reduced direct oversight.

To understand the potential risks of digital tracking, it is essential to consider how adolescents perceive its use (Darling et al. 2008; Smetana 2024). In our sample of 525 adolescents aged 11 to 18 years, the majority found it an acceptable parenting practice. Location tracking was the least accepted, however, suggesting that parents may more frequently track domains that adolescents find more acceptable. Additionally, many adolescents seemed aware that they were being tracked, and many even thought their parents engaged in tracking *more* frequently than their parents indicated. Hence, the present findings suggest that many adolescents are aware of and seem to accept this modern form of passive parental monitoring.

### 4.2 Digital Tracking Is More Common in Some Households

To better understand in which families digital tracking occurs more frequently, we examined static demographic factors. Consistent with Burnell et al. (2023), no parent demographics were linked to the frequency of location tracking. This suggests that location tracking may be used among diverse families, reflecting a common parental concern about adolescents' activities outside the home. However, we did find several demographic associations for school and financial tracking —domains that have received little empirical attention to date, ranging from small to moderate effects (Gignac and Szodorai 2016). That is, younger parents reported more frequent tracking of school performance and financial activities, possibly reflecting greater technological familiarity, suggesting that future generations of parents may rely even more on digital tracking tools. Furthermore, mothers and lower‐educated parents specifically tracked school performance more often, suggesting that mothers may carry a greater mental load and feel more pressure to be involved in their children's schooling (Venard et al. 2024), whereas lower‐educated parents may track school performance more intensively to help their child surpass their own educational attainment (Wuyts et al. 2015).

Tracking frequency was also associated with adolescents' demographic factors, with effect sizes ranging from small to large. Adolescents in lower educational tracks were tracked more frequently across all domains, which might reflect that more at‐risk adolescents are monitored more closely (Racz and McMahon 2011). As much prior research has focused on parental education rather than adolescent education (e.g., Davis et al. 2024), this finding offers an important insight to unravel for future research. Moreover, given that lower‐educated adolescents were underrepresented in our samples (Central Bureau for Statistics 2024), the prevalence of digital tracking among Dutch parents reported in this study is thus likely an underestimation. Furthermore, male adolescents were tracked more frequently in the school domain, which may reflect boys' generally less positive school attitudes and engagement compared to girls (Van Houtte 2004), prompting parents to track boys' school performance more closely. Additionally, younger adolescents experienced more school‐related tracking, whereas older adolescents experienced more location tracking. Hence, parents' motivations to track may vary according to development, with parents first being more concerned about school performance and later shifting toward concerns about adolescents' whereabouts as they start to spend more time outside the home.

### 4.3 Digital Tracking Is Linked to Parents' Insecurities and Need for Control

Parents' worries and anxiety about their child's safety and functioning might be a key driver of their digital tracking use (Davis et al. 2024; Wood et al. 2003). The present study found, however, no evidence that parents with higher levels of general anxiety or neuroticism engage in more frequent tracking. However, parents characterized by higher intolerance of uncertainty, a strong need for control and predictability (Gibb et al. 2023), and those holding more dangerous worldviews (Zimmermann et al. 2024), reported more frequent digital tracking across domains, though these associations were small in magnitude. In addition, separation anxiety was related to school performance and location tracking, suggesting that some parents may use tracking to reassure themselves about their child's whereabouts and functioning. School‐related tracking was specifically linked to parenting perfectionism. Parents might monitor their child's school performance as part of their own striving to be the “perfect parent” ‐ staying informed about grades, schedules, and homework enables them to provide the most optimal level of support. Relatedly, parents who track more frequently were perceived by both themselves and their adolescents as more overprotective. Digital tracking might therefore be viewed as a modern extension of parents' overprotection, used to minimize perceived risks, in line with the review of Davis et al. (2024), who identified parental worry and safety concerns as primary motives for location tracking. Our results suggest that similar anxiety‐driven mechanisms may extend beyond location tracking to school and financial tracking as well.

Taken together, the present findings suggest that parents' frequency to digital track their adolescents is related to their own insecurities, fear, and need for control. Longitudinal, within‐person research is needed (Hamaker 2025) to disentangle whether frequent tracking causes, results from, or exacerbates these parental insecurities over time (Gabriels 2016). Does digital tracking provide the reassurance parents are seeking, or does it fuel their worries? If digital tracking leads parents to become more overprotective and anxious, this raises concerns for future generations of parents as well as for adolescents (Zhang and Ji 2024).

### 4.4 Digital Tracking Not Linked to Adolescent Well‐Being

The present study tapped into the concerns that the shift from interaction‐based to data‐driven parental monitoring may undermine adolescents' privacy, autonomy, and well‐being (Davis et al. 2024; Gabriels 2016). However, the frequency of digital tracking was not robustly associated with adolescents' autonomy frustration, depressive, and general anxiety symptoms, or perceived school pressure. These null findings are consistent with Burnell et al. (2023), who found no associations between location tracking frequency and adolescent internalizing or externalizing problems. One possible explanation might be that parental tracking behavior may be more parent‐driven than child‐driven. Additionally, today's adolescents grow up in a world where continuous connectivity is omnipresent (Dereymaeker et al. 2025; Janssen et al. 2025; Marciano 2022), and many might even track their parents in return. With digital tracking potentially being an increasingly normalized and accepted parenting practice, digital tracking might not be experienced as a privacy violation or a threat to autonomy. Future longitudinal, within‐family research is needed to study whether, for whom, when, and under what conditions digital tracking is harmful or beneficial (Keijsers et al. 2026).

### 4.5 Strengths, Limitations, and Future Directions

Apart from the cross‐sectional design and the relatively small replication sample from 2019 (*N* = 175), several limitations need to be mentioned. The first set of limitations concerns the measurement of parental digital tracking. The measure of digital tracking was self‐developed and has not yet been validated or used in other studies. Moreover, tracking frequency was based on self‐reports, which may be prone to underestimation. Tracking was also treated as a static behavior, though its frequency likely fluctuates depending on both parental and adolescent factors (Pelham et al. 2024). Future research could benefit from using more fine‐grained and objective methods to capture digital family life (Keijsers et al. 2022). Additionally, not all relevant domains of digital monitoring were assessed (Beyens et al. 2024; Modecki et al. 2022), such as phone use, which might be perceived by adolescents as more private than domains like school or finances.

A second limitation is that the data were solely collected from Dutch families, limiting the generalizability of the findings to other countries. For instance, the use of online school portals may be less common or function differently in other countries. Large, representative cross‐national studies are needed to improve the understanding of the prevalence and nature of parental digital tracking globally.

## 5 Conclusion

Digital tracking has become a common and accepted practice, with nearly all Dutch parents tracking their adolescent in at least one domain ‐ school, location, or finances. Tracking frequency appears to be linked to parental characteristics. Frequent tracking might be a digital form of overprotection, motivated by parents' struggles with uncertainty, fear, and a need for control. However, given the cross‐sectional nature of the data, longitudinal research is crucial to study whether digital tracking provides parents comfort and reassurance, or whether it reinforces their fears and insecurities, ultimately harming their own well‐being, but most likely also their adolescents'.

## Author Contributions

**Savannah Boele:** conceptualization, investigation, writing – original draft, formal analysis, data curation, methodology. **Anne Bülow:** conceptualization, writing – review and editing, methodology, investigation. **Loes Keijsers:** conceptualization, writing – review and editing, funding acquisition, methodology.

## Ethics Statement

The procedure for the data collection has been reviewed and approved by the Department of Psychology, Education, and Child Studies (DPECS) Research Ethics Review Committee at Erasmus University Rotterdam on the 23rd of August 2024 (ETH2324‐0156). All participants provided written informed consent. For adolescent participants under the age of 16, informed consent was also obtained from their parents.

## Conflicts of Interest

The authors declare no conflicts of interest.

## Supporting information


Supporting File

## Data Availability

The data that support the findings of this study are openly available in OSF at https://osf.io/znu9w).
