# Supplementary material for: Parental Digital Tracking of Adolescents' School Performance, Location, and Finances: Widespread, Often Accepted, and Linked to Overprotection
Source: J Adolesc. 2026 Jul 20;98(7):2478–90. doi: 10.1002/jad.70229 (PMC13643634; doi:10.1002/jad.70229)
Supplement: Supplementary file 1 [file JAD-98-2478-s001.docx]

**Supporting Information A – Descriptive Statistics and Correlations**

**Table A1**
Overview of Demographic Characteristics of the Sample of Study 1

| **Characteristic** | **Category** | **%** |
| --- | --- | --- |
| ***Adolescents* *(N* = 525)** |  |  |
| Age (in years) | *M* = 14.1 (*SD* = 1.6, range = 11–18) | - |
| Sex | Female | 52.6% |
|  | Male | 45.7% |
|  | Other | 0.2% |
| Country of birth | Netherlands | 95.4% |
| Nationality | Dutch | 95.4% |
| Living area | Rural | 52.2% |
|  | Urban | 46.3% |
| Siblings | Has siblings | 89.9% |
|  | One sibling | 53.1% |
|  | Two siblings | 28.2% |
| Birth order | Oldest | 48.2% |
|  | Second oldest | 30.7% |
| Educational track | (pre-)vocational | 18.1% |
|  | Higher general education | 32.8% |
|  | Pre-university | 43.2% |
|  | Mixed secondary track | 3.2% |
|  | Applied university | 1.0% |
| Missing data of demographic information | - | 1.5% |
| ***Parents* (N = 525)** |  |  |
| Age (in years) | *M* = 46.7 (*SD* = 4.7, range = 31–61) |  |
| Relation to adolescent | Biological mother | 81.1% |
|  | Biological father | 17.9% |
|  | Adoptive mother | 0.6% |
|  | Stepmother | 0.2% |
|  | Stepfather | 0.2% |
| Caregiving role | Shared equally | 67.2% |
|  | Primary caregiver | 32.2% |
|  | Secondary caregiver | 0.6% |
| Marital status | With the biological parent | 79.6% |
|  | New partner | 11.8% |
|  | Single | 7.1% |
|  | Widowed | 1.5% |
| Country of birth | Netherlands | 97.0% |
| Nationality | Dutch | 96.8% |
| Educational level | Secondary school | 3.4% |
|  | Vocational training | 18.5% |
|  | Applied university | 44.4% |
|  | University | 33.1% |
| Missing data of demographic information | - | 0.6% |

**Table A2**
*Descriptive Statistics of all Study Variables in Sample 1 (N = 525)*

| **Variables** | ***M*** | ***SD*** | **Range** | **Skewness** | ***n*** |
| --- | --- | --- | --- | --- | --- |
| ***Parent-reported*** |  |  |  |  |  |
| 1. Digitally tracking school | 3.3 | 0.9 | 1-5 | -0.5 | 525 |
| 2. Digitally tracking location | 2.4 | 1.3 | 1-5 | 0.3 | 525 |
| 3. Digitally tracking finances | 2.6 | 0.9 | 1-5 | 0.0 | 525 |
| 4. Parental emotional support | 3.5 | 0.5 | 2.4-4.7 | 0.1 | 521 |
| 5. Parental autonomy support | 4.1 | 0.4 | 2.7-5.0 | -0.3 | 521 |
| 6. Parental overprotection | 2.2 | 0.4 | 1.1-3.4 | 0.1 | 521 |
| 7. Parental anxiety symptoms | 1.5 | 0.5 | 1.0-3.9 | 1.7 | 521 |
| 8. Parental neuroticism | 2.5 | 0.6 | 1.0-4.7 | 0.4 | 525 |
| ***Adolescent-reported*** |  |  |  |  |  |
| 9. Parental emotional support | 3.8 | 0.6 | 1.3-5.0 | -0.7 | 515 |
| 10. Parental autonomy support | 4.0 | 0.5 | 2.1-5.0 | -0.4 | 515 |
| 11. Parental overprotection | 2.3 | 0.4 | 1.1-3.9 | 0.09 | 515 |
| 12. Adolescent legitimacy beliefs of digital school tracking | 2.7 | 0.6 | 1.0-3.0 | -1.5 | 515 |
| 13. Adolescent legitimacy beliefs of digital location tracking | 2.1 | 0.8 | 1.0-3.0 | -0.2 | 515 |
| 14. Adolescent legitimacy beliefs of digital financial tracking | 2.6 | 0.6 | 1.0-3.0 | -1.3 | 515 |
| 15. Adolescent autonomy frustration | 2.6 | 0.9 | 1.0-5.0 | 0.4 | 515 |
| 16. Adolescent depressive symptoms | 1.9 | 0.6 | 1.0-4.0 | 0.7 | 514 |
| 17. Adolescent anxiety symptoms | 1.8 | 0.5 | 1.0-3.0 | 0.4 | 514 |
| 18. Adolescent school pressure | 2.7 | 1.5 | 1.0-5.0 | 0.2 | 408 |

**Table A3**
*Bivariate Pearson Correlations between all Study Variables (N = 525)*

| **Variables** | **1.** | **2.** | **3.** | **4.** | **5.** | **6.** | **7.** | **8.** | **9.** | **10.** | **11.** | **12.** | **13.** | **14.** | **15.** | **16.** | **17.** |
| --- | --- | --- | --- | --- | --- | --- | --- | --- | --- | --- | --- | --- | --- | --- | --- | --- | --- |
| ***Parent-reported variables*** |  |  |  |  |  |  |  |  |  |  |  |  |  |  |  |  |  |
| 1. Digitally tracking school | - |  |  |  |  |  |  |  |  |  |  |  |  |  |  |  |  |
| 2. Digitally tracking location | **.20***** | - |  |  |  |  |  |  |  |  |  |  |  |  |  |  |  |
| 3. Digitally tracking finances | **.29***** | .**26***** | - |  |  |  |  |  |  |  |  |  |  |  |  |  |  |
| 4. Parental emotional support | **.18***** | **.12**** | **.09*** | - |  |  |  |  |  |  |  |  |  |  |  |  |  |
| 5. Parental autonomy support | -.02 | .00 | -.06 | .25*** | - |  |  |  |  |  |  |  |  |  |  |  |  |
| 6. Parental overprotection | **.18***** | **.18***** | **.15***** | **.16***** | **-.30***** | - |  |  |  |  |  |  |  |  |  |  |  |
| 7. Parental anxiety symptoms | .00 | .06 | -.01 | -.08 | -.06 | **.21***** | **-** |  |  |  |  |  |  |  |  |  |  |
| 8. Parental neuroticism | .04 | .08 | .00 | -.05 | **-.12**** | **.34***** | **.57***** | **-** |  |  |  |  |  |  |  |  |  |
| ***Adolescent-reported variables*** |  |  |  |  |  |  |  |  |  |  |  |  |  |  |  |  |  |
| 9. Parental emotional support | .01 | .03 | -.01 | **.33***** | **.13**** | -.01 | -.03 | -.06 | - |  |  |  |  |  |  |  |  |
| 10. Parental autonomy support | **-.10*** | -.01 | **-.12**** | **.14**** | **.22***** | -.07 | -.07 | -.03 | **.53***** | - |  |  |  |  |  |  |  |
| 11. Parental overprotection | **.21***** | .06 | **.16***** | .08 | -.07 | **.25***** | .01 | **.10*** | **-.17***** | **-.33***** | - |  |  |  |  |  |  |
| 12. Legitimacy beliefs school tracking | .06 | .07 | -.07 | **.11*** | .05 | **.11*** | .05 | -.03 | **.27***** | **.23***** | **-.11*** | - |  |  |  |  |  |
| 13. Legitimacy beliefs location tracking | .01 | **.26***** | -.01 | **.16***** | .05 | .02 | .05 | .01 | **.28***** | **.18***** | **-.10*** | **.24***** | - |  |  |  |  |
| 14. Legitimacy beliefs financial tracking | -.01 | .04 | .00 | .07 | .04 | .04 | .05 | .01 | **.28***** | **.22***** | **-.18***** | **.34***** | **.32***** | - |  |  |  |
| 15. Adolescent autonomy frustration | .00 | -.02 | -.06 | -.06 | -.01 | .03 | .08 | .04 | **-.26***** | **-.25***** | **.21***** | **-.14**** | **-.18***** | **-.17***** | - |  |  |
| 16. Adolescent depressive symptoms | -.08 | .05 | -.01 | **-.13**** | -.08 | .08 | **.15***** | **.12**** | **-.31***** | **-.27***** | **.12**** | **-.18***** | **-.18***** | **-.23***** | **.42***** | - |  |
| 17. Adolescent anxiety symptoms | -.08 | .03 | **-.09*** | -.05 | .01 | .05 | **.12**** | **.10*** | **-.21***** | **-.14**** | **.14**** | **-.09*** | **-.16***** | **-.19***** | **.41***** | **.75***** | - |
| 18. Adolescent school pressure | .01 | .02 | -.07 | -.08 | -.03 | .06 | **.12*** | .07 | -.04 | .04 | **.10*** | -.01 | **-.10*** | -.04 | **.19***** | **.20***** | **.28***** |

*Note*. Significant correlations are in bold.

* *p* < .05, ** *p* < .01, *** *p* < .001

**Supporting Information B**

**Figure B1**

*Patterns of Parental Digital Tracking Across Domains*

*Note*. Sometimes = several times a year *or* month. Often = several times a week *or* day.

**Supporting Information C– Sample and Results of Study 2**

**Participants**

More information about this completed study can be found in [https://osf.io/e2jzk]. Parent-adolescent dyads were included in the current study if data was available of the self-reported outcome ‘parental digital tracking’. The analytical sample therefore included 175 dyads. The adolescents had a mean age of 14.1 years (*SD* = 1.5, range = 12–17), with 69.1% identifying as female and 30.9% as male. The majority were born in the Netherlands (97.1%). Adolescents’ educational tracks ranged from pre-vocational or vocational training (17.1%), higher general secondary education (25.7%), and pre-university education (52.6%) to mixed secondary tracks (4.6%) and applied university (1.0%).

The participating parents had a mean age of 46.8 years (*SD* = 5.2, range = 36–76) and were the biological mother (81.7%) or biological father (18.3%) of the adolescent. Most parents were born in the Netherlands (90.3%). Regarding education, for 13.1% highest educational level was completing elementary or secondary school, 35.4% vocational training, 32.0% applied university, and 19.4% university.

**Table C1**
*Descriptive Statistics of all Study Variables in Sample 2 (N = 175)*

| **Variables** | ***M*** | ***SD*** | **Range** | **Skewness** | ***n*** |
| --- | --- | --- | --- | --- | --- |
| ***Parent-reported*** |  |  |  |  |  |
| 1. Digitally tracking school | 3.3 | 1.0 | 1-5 | -0.6 | 175 |
| 2. Digitally tracking location | 1.7 | 1.2 | 1-5 | -0.1 | 175 |
| 3. Digitally tracking finances | 2.6 | 1.0 | 1-4 | -0.2 | 172 |
| 4. Parental emotional support | 4.5 | 0.5 | 2.0-5.0 | -1.5 | 175 |
| 5. Parental autonomy support | 4.1 | 0.5 | 2.5-5.0 | 0.1 | 175 |
| 6. Parental overprotection parenting | 3.5 | 0.8 | 1.0-5.4 | -0.5 | 136 |
| 7. Parental anxiety symptoms | 1.7 | 0.5 | 1.0-3.8 | 1.0 | 174 |
| 8. Parental neuroticism | 2.3 | 0.8 | 1.0-5.0 | 0.6 | 174 |
| ***Adolescent-reported*** |  |  |  |  |  |
| 9. Parental emotional support | 4.6 | 0.5 | 2.3-5.0 | -1.4 | 174 |
| 10. Parental autonomy support | 4.3 | 0.6 | 2.3-5.0 | -0.6 | 174 |
| 11. Parental overprotection | 3.3 | 0.8 | 1.1-5.1 | -0.2 | 115 |
| 12. Adolescent legitimacy beliefs of digital school tracking | 2.7 | 0.6 | 1.0-3.0 | -1.5 | 173 |
| 13. Adolescent legitimacy beliefs of digital location tracking | 1.7 | 0.8 | 1.0-3.0 | 0.6 | 173 |
| 14. Adolescent legitimacy beliefs of digital financial tracking | 2.5 | 0.6 | 1.0-3.0 | -0.8 | 173 |
| 15. Adolescent autonomy frustration | 3.6 | 0.8 | 1.0-5.0 | -0.6 | 139 |
| 16. Adolescent depressive symptoms | 1.8 | 0.6 | 1.0-3.7 | 0.9 | 173 |
| 17. Adolescent anxiety symptoms | 1.7 | 0.5 | 1.0-3.0 | 0.6 | 173 |
| 18. Adolescent school pressure | 2.5 | 0.9 | 1.0-5.0 | 0.5 | 173 |

**Table C2**
*Bivariate Pearson Correlations between all Study Variables in Sample 2 (N = 175)*

| **Variables** | **1.** | **2.** | **3.** | **4.** | **5.** | **6.** | **7.** | **8.** | **9.** | **10.** | **11.** | **12.** | **13.** | **14.** | **15.** | **16.** | **17.** |
| --- | --- | --- | --- | --- | --- | --- | --- | --- | --- | --- | --- | --- | --- | --- | --- | --- | --- |
| ***Parent-reported*** |  |  |  |  |  |  |  |  |  |  |  |  |  |  |  |  |  |
| 1. Digitally tracking school | - |  |  |  |  |  |  |  |  |  |  |  |  |  |  |  |  |
| 2. Digitally tracking location | .14 | - |  |  |  |  |  |  |  |  |  |  |  |  |  |  |  |
| 3. Digitally tracking finances | **.24**** | .**21**** | - |  |  |  |  |  |  |  |  |  |  |  |  |  |  |
| 4. Parental emotional support | .09 | -.02 | .01 | - |  |  |  |  |  |  |  |  |  |  |  |  |  |
| 5. Parental autonomy support | **-.19*** | -.03 | -.15 | **.28***** | - |  |  |  |  |  |  |  |  |  |  |  |  |
| 6. Parental overprotection parenting | **.22***** | .11 | **.22*** | .09 | **-.36***** | - |  |  |  |  |  |  |  |  |  |  |  |
| 7. Parental anxiety symptoms | .14 | .06 | .08 | **-.27***** | -.05 | -.01 | **-** |  |  |  |  |  |  |  |  |  |  |
| 8. Parental neuroticism | **.23**** | .-.02 | .02 | **-.25**** | .08 | .09 | **.58***** | **-** |  |  |  |  |  |  |  |  |  |
| ***Adolescent-reported*** |  |  |  |  |  |  |  |  |  |  |  |  |  |  |  |  |  |
| 9. Parental emotional support | .05 | -.03 | -.04 | **.30***** | .05 | .03 | **-.23**** | -.12 | - |  |  |  |  |  |  |  |  |
| 10. Parental autonomy support | **-.20**** | -.12 | -.04 | **.17**** | **.31***** | -.11 | **-.23**** | **-.16*** | **.41***** | - |  |  |  |  |  |  |  |
| 11. Parental overprotection | **.22**** | -.08 | .18 | .06 | -.06 | **.22*** | -.04 | .03 | -.08 | -.11 | - |  |  |  |  |  |  |
| 12. Legitimacy beliefs school tracking | -.01 | .06 | -.02 | **.24*** | .14 | .06 | **-.20**** | -.07 | **.33***** | **.25***** | -.05 | - |  |  |  |  |  |
| 13. Legitimacy beliefs location tracking | .09 | **.35***** | -.04 | **.19**** | -.06 | .06 | .05 | -.03 | **.34***** | .03 | -.15 | **.24**** | - |  |  |  |  |
| 14. Legitimacy beliefs financial tracking | **.19*** | -.03 | .14 | .18* | -.12 | .11 | -.07 | -.01 | **.19*** | .08 | .07 | **.39***** | **.28***** | - |  |  |  |
| 15. Adolescent autonomy frustration | -.06 | .00 | .01 | .05 | .05 | -.09 | -.14 | -.09 | **.20*** | **.22*** | -.07 | .08 | .10 | **.**15 | - |  |  |
| 16. Adolescent depressive symptoms | -.01 | .01 | .06 | **-.19**** | .11 | -.05 | **.24**** | .10 | **-.45***** | **-.30***** | .04 | **-.27***** | **-.25***** | **-.33***** | **-.22**** | - |  |
| 17. Adolescent anxiety symptoms | -.12 | .08 | .06 | -.07 | .14 | .03 | **.17*** | .09 | **-.28***** | **-.17*** | -.08 | **-.17*** | -.07 | **-.21**** | -.12 | **.73***** | - |
| 18. Adolescent school pressure | -.01 | .06 | .00 | -.01 | .**24**** | -.09 | .14 | .**15*** | **-.29***** | .12 | .05 | -.14 | -.14 | **-.21**** | **-.25**** | **.54***** | **.62***** |

*Note*. Significant correlations are in bold. * p < .05, ** p < .01, *** p < .001

**Table C3**

*Results of the Exploratory Factor Analysis and Inter-Item Correlations of the Digital Tracking Items (α = .41) in Sample 2 (N = 175)*

| **Item No.** | **Item** | **Factor loadings** | ***h*^2^** | **Inter-item correlations** | |
| --- | --- | --- | --- | --- | --- |
|  |  |  |  | **1.** | **2.** |
| 1. | How often do you digitally monitor school-related information (e.g., grades, homework, or attendance)? | 0.40 | 0.16 | - | **-** |
| 2. | How often do you digitally track your child's location (e.g., via Find My iPhone or a GPS tracker)? | 0.35 | 0.12 | 0.14 | - |
| 3. | How often do you check the money in your child's bank account (e.g., via a banking app)? | 0.59 | 0.35 | 0.24* | 0.21* |

*Note*. *h*^2^ = communalities, representing the proportion of each item’s explained variance by the factor. * *p* < .01

**Table C4**

*Sample Frequencies of Parent-Reported Digital Tracking (N = 175)*

| **Response** | **School** | **Location** | **Finances** |
| --- | --- | --- | --- |
| 1. Never | 10 (5.7%) | **116 (66.3%)** | 28 (16.0%) |
| 2. Several times a year | 26 (14.9%) | 17 (9.7%) | 43 (24.6%) |
| 3. Several times a month | 47 (26.9%) | 16 (9.1%) | **71 (40.6%)** |
| 4. Several times a week | **79 (45.1%)** | 24 (13.7%) | 30 (17.1%) |
| 5. Several times a day | 13 (7.4%) | 2 (1.1%) | 0 (0%) |

*Note*. Bold values indicate modus (i.e., most chosen answer).

**Table C5**

*Results of Pre-Registered Robust Regression Predicting Frequency of Digital Tracking (N = 175)*

|  | **Digital Tracking Behaviors** | | |
| --- | --- | --- | --- |
|  | **School** | **Location** | **Finances** |
|  | **β** | **β** | **β** |
| **Model 1: Parent characteristics** |  |  |  |
| Age | -.15 | -.13 | .05 |
| Sex (0 = female (81.7%), 1 = male (18.3%)) | -.33 | .24 | -.05 |
| Educational level (0 = low and middle (48.6%),  1 = high (50.6%)) | -.27 | .03 | -.25 |
| **Model 2: Adolescent characteristics** |  |  |  |
| Age | -.26** | -.02 | .10 |
| Sex (0 = female (69.1%), 1 = male (30.9%)) | .28 | .06 | .24 |
| Educational track (0 = low and middle (47.4%),  1 = high (52.6%)) | -.04 | -.13 | -.42* |
| **Model 3: Parent well-being and personality** |  |  |  |
| Parental anxiety (α = .83) | .20* | -.08 | -.04 |
| Parental neuroticism (α = .73) | .03 | .10 | .11 |
| **Model 4: Parent-reported parenting** |  |  |  |
| Emotional support (α = .83) | .18 | -.08 | .04 |
| Autonomy support (α = .89) | -.17 | .03 | -.08 |
| Overprotective parenting (α = .79) | .14 | .09 | .21* |
| **Model 5: Adolescent-reported parenting** |  |  |  |
| Emotional support (α = .83) | .19 | .08 | .00 |
| Autonomy support (α = .86) | -.14 | -.13 | .04 |
| Overprotective parenting (α = .72) | .22* | -.08 | .24* |
| **Model 6: Adolescent well-being** |  |  |  |
| Anxiety symptoms (α = .89) | -.15 | .13 | -.02 |
| Depressive symptoms (α = .87) | -.09 | -.14 | .06 |
| Autonomy satisfaction (α = .72) | -.06 | -.01 | .03 |
| **Model 7: Adolescent school pressure** |  |  |  |
| Adolescent school pressure^1^ (α = .72) | -.03 | - | - |

*Note*. Note. Educational level parents: low = secondary school; middle = vocational training; high = (applied) university). Educational tracks adolescents: low = pre-vocational training; middle = higher general secondary education or vocational training; high = pre-university or (applied) university.

* p < .05, ** p < .01, *** p < .001

**Table C6**

*Sample Frequencies of Adolescents’ Legitimacy Beliefs of Digital Tracking (N = 173)*

| **Response** | **School** | **Location** | **Finances** |
| --- | --- | --- | --- |
| 1. Not okay | 7 (4.1%) | **84 (48.6%)** | 11 (6.4%) |
| 2. Somewhat okay | 41 (23.7%) | 59 (34.1%) | 67 (38.7%) |
| 3. Totally okay | **125 (72.3%)** | 30 (17.3%) | **95 (54.9%)** |

*Note*. Bold values indicate modus (i.e., most chosen answer).

**Supporting Information D – Scales and Correlations of Exploratory Scales**

**Measures**

***Parental Separation Anxiety***

Parents’ separation anxiety was measured with the Parents of Adolescents Separation Axniety Scale (PASAS; Hock et al., 2001; Wutys et al., 2017). The PASAS consists of six items, such as “It hurts my feelings when my teenager takes his/her problems to a good friend instead of to me.”, which are responded on a scale from 1 (*totally disagree*) to 5 (*totally agree*).

***Parental Threat Perception***

Parents’ threat perception was measured with the ‘World Out There’ scale (Gurland & Grolnick et al., 2005), which includes 10 items across three subscales: worry, scarcity, and instability. Participants rated statements such as “It makes me nervous to think about all the dangers kids are exposed to these days” on a 5-point Likert scale ranging from 1 (*strongly disagree*) to 5 (*strongly agree*).

***Parents’ Dangerous Worldview***

The ‘Social Worldview Scale Revised’ was used to measure parents’ worldviews (Perry et al., 2013), consisting of 6 items. An example item is “There are many dangerous people in our society who will attack someone out of pure meanness, for no reason at all.”. The 5-point Likert scale ranges from 1 (*strongly disagree*) to 5 (*strongly agree*).

***Parenting Perfectionism***

Parenting perfectionism was measured using the Multidimensional Parenting Perfectionism Questionnaire (MPPQ; Snell et al., 2005). The MPPQ consists of 12 items across two subscales: self-oriented parenting perfectionism (e.g., “set very high standards for myself as a parent”) and societal prescribed perfectionism (e.g., “Only if I am “perfect” as a parent will society consider me to be a good parent”). The items are rated on a 5-point Likert scale from 1 (*not at all characteristic of me*) to 5 (*very characteristic of me*).

***Parents’ Intolerance for Uncertainty***

Parents’ intolerance for uncertainty was measured with the Intolerance of Uncertainty Scale 12-item version (IUS-12; Carleton et al., 2007; Helsen et al., 2013). The 12-item scale consists of two subscales: prospective anxiety (7 items, e.g., “ Unforeseen events upset me greatly.”) and inhibitory anxiety (5 items, e.g., “Uncertainty keeps me from living a full life.”). The items are rated on a 5-point Likert scale from 1 (*not at all representative*) to 5 (*completely representative*).

***Parents’ Optimism***

Parental optimism was measured with the Revised Life Orientation Test (LOT-R; Scheier et al., 1994; Klooster et al., 2010). The LOT-R consists of 10 items, of which 4 items are filler items, all rated on a 5-point Likert scale from 1 (*strongly disagree*) to 5 (*strongly agree*). An example item is “In uncertain times, I usually expect the best”.

***Adolescents’ Separation Anxiety***

Separation anxiety of the adolescent was measured with the Youth Anxiety Measure for DSEM-5 (YAM-5-I; Muris et al., 2017; Simon & Muris, 2006). The YAM-5-I consists of six items, such as “I am afraid to go anywhere without my parent(s)”, which are rated on a scale from 0 (*never*) to 3 (*always*).

***Adolescents’ Intolerance for Uncertainty***

Similar as for the parents, adolescents intolerance for uncertainty was measured with the Intolerance of Uncertainty Scale 12-item version (IUS-12; Carleton et al., 2007; Helsen et al., 2013). The 12-item scale consists of two subscales: prospective anxiety (7 items, e.g., “ Unforeseen events upset me greatly.”) and inhibitory anxiety (5 items, e.g., “Uncertainty keeps me from living a full life.”). The items are rated on a 5-point Likert scale from 1 (*not at all representative*) to 5 (*completely representative*).

***Adolescents’ Perfectionism***

The Child-Adolescent Perfectionism Scale (CAPS) short form was used to measure adolescents’ trait perfectionism (Flett et al., 2016; Bento et al., 2020). The short form of the CAPS includes two subscales: self-oriented (4 items; e.g., “ I try to be perfect in everything I do”) and socially prescribed perfectionism (5 items; e.g., “I feel that people ask too much of me.”). The items are rated on a 5-point Likert scale from 1 (*not true at all of me*) to 5 (*very true of me*).

**Table D1**

*Bivariate correlations between exploratory parental correlates*

| **Variables** | **1.** | **2.** | **3.** | **4.** | **5.** |
| --- | --- | --- | --- | --- | --- |
| ***Parent-reported data*** |  |  |  |  |  |
| 1. Separation anxiety | - |  |  |  |  |
| 2. Threat perception | .28*** | - |  |  |  |
| 3. Dangerous worldview | .24*** | .60*** | - |  |  |
| 4. Parenting perfectionism | .31*** | .22*** | .20** | - |  |
| 5. Intolerance for uncertainty | .39*** | .33*** | .30*** | .45*** | - |
| 6. Optimism | -.27*** | .38*** | -.40*** | -.27*** | -.46*** |

*Note*. *** *p* < .001

**Table D2**

*Bivariate correlations between exploratory adolescent correlates*

| **Variables** | **1.** | **2.** |
| --- | --- | --- |
| ***Adolescent-reported data*** |  |  |
| 1. Separation anxiety | - |  |
| 2. Intolerance for uncertainty | .22*** | - |
| 3. Perfectionism | .36*** | .44*** |

*Note*. *** *p* < .001
